# A Multicenter Retrospective Outcomes Analysis of Patients with Localized Synovial Sarcoma

**DOI:** 10.1158/2767-9764.CRC-25-0652

**Published:** 2026-06-03

**Authors:** Stefano Testa, Maggie Yuxi Zhou, Brian C. Schulte, Victoria E. Wang, Katie Kenny, Austine Peng, Marta Lohman, Anupam M. Desai, Bruno Bockorny, Ali Baghian, Daniel R. Schmidt, Nam Q. Bui, Minggui Pan, Kristen N. Ganjoo, Varun Monga

**Affiliations:** 1 Division of Hematology and Oncology, Beth Israel Deaconess Medical Center, Boston, Massachusetts.; 2 Division of Oncology, Department of Medicine, Stanford University, Stanford, California.; 3 UCSF Helen Diller Family Comprehensive Cancer Center, San Francisco, California.

## Abstract

To identify clinicopathologic factors associated with outcomes in patients with localized synovial sarcoma (SS). We retrospectively reviewed 248 patients with localized SS treated across three US quaternary medical centers between 2000 and 2024. Demographics, tumor characteristics, and treatments, including perioperative chemotherapy and radiotherapy (RT), were assessed. Disease‐free survival (DFS) and overall survival (OS) were estimated via Kaplan–Meier analysis, and Cox regression was used for univariable and multivariable analysis, including significant univariate factors and confounders such as age and tumor size. Median follow-up was 5.2 years; median age at diagnosis was 35 years (IQR, 26–46). Median DFS was 110.3 months. Perioperative chemotherapy was given to 150 (60%) patients, whereas perioperative RT was given to 173 (69.8%) patients. Multivariable analysis showed that tumor size correlated with worse DFS (*P* < 0.001) and OS (*P* < 0.001), whereas a location in the chest wall, trunk, or extremities correlated with improved DFS (*P* = 0.006). Percent tumor necrosis was associated with worse OS (*P* = 0.04), whereas R0 (*P* = 0.002) resection margins correlated with improved OS. Perioperative RT or chemotherapy did not correlate with OS or DFS in the whole cohort, though patients with tumor size ≥10 cm (*n* = 47) showed worse OS when receiving perioperative chemotherapy only as opposed to combined perioperative chemotherapy and RT (*P* = 0.006). Tumor size, location, margin status, and tumor necrosis are independent prognostic factors in localized SS. Combined perioperative chemotherapy and RT correlated with improved OS in patients with tumors larger than 10 cm.

**Significance:**

SS is a rare, aggressive malignancy with limited perioperative data. In this multi‐institutional study of 248 patients with localized disease, tumor size, location, and margin status predicted recurrence and survival. Combined perioperative chemotherapy and RT benefited patients with larger tumors but not across the overall cohort, supporting risk-adapted management.

## Introduction

Soft tissue sarcomas (STS) are a group of rare and heterogeneous mesenchymal neoplasms that together account for approximately 1% of all cancers, with a relatively higher incidence among pediatric and young adult patients in whom they account for around 8% of all malignant tumors (1–4). Among STSs, synovial sarcoma (SS) represents a particularly aggressive subtype accounting for 5% to 10% of STS cases, which tends to affect mostly young adults with a mean age at diagnosis of 39 years and no difference in incidence between males and females (5). Prior retrospective studies and multicenter analyses focused on SS have shown that outcomes are heterogeneous and influenced by clinicopathologic features, including tumor size, site, surgical margin status, grade, mitotic activity, necrosis, and, in some cohorts, patient age and extent of local invasion, supporting risk-adapted perioperative management strategies (6–8).

The cornerstone of management of patients with localized SS is surgical resection with negative margins (9). Although preoperative radiation remains the standard of care for tumors ≥5 cm and grade ≥2, perioperative chemotherapy is also used for patients with larger, high-risk tumors (10, 11). The role of external-beam radiotherapy (EBRT) in localized extremity STS to improve local control is well documented in both randomized studies (12, 13) and retrospective reviews (14). Intraoperative radiotherapy (IORT), often combined with EBRT, is typically reserved for patients with primary tumors in challenging locations and has been associated with favorable local control in retrospective series of extremity STS, although data are nonrandomized (15, 16). The role of perioperative chemotherapy in localized SS also remains controversial. Retrospective analyses focused on SS have suggested potential benefit in selected high-risk patients treated with ifosfamide-based regimens (17), whereas other multicenter retrospective studies have not demonstrated a clear survival advantage after adjustment for prognostic factors. Overall, significant variability in real-world clinical practice exists around perioperative management of patients with high-risk localized SS. In this study, we analyzed a large cohort of patients with localized SS treated at three different sarcoma centers in the United States. Our data provide insight into treatment practices for patients with localized SS and elucidates the role of multimodal perioperative management of patients with SS.

## Materials and Methods

### Data collection

Patient cohorts were independently determined at each participating institution and included pathologically and molecularly confirmed diagnosis of SS treated between 2000 and 2024. Individuals with metastatic disease, either biopsy-proven or suspected based on imaging, were excluded from the study. Patient data were collected through retrospective review of electronic health records. Information collected included baseline patient characteristics and specifically age at diagnosis, gender, ethnicity, date of diagnosis, SS histologic subtype, tumor size, and anatomic location, *SS18* gene fusion partner, and tumor stage. Treatment-related variables measured included posttreatment pathologic necrosis, neoadjuvant and adjuvant chemotherapy regimens and radiotherapy (RT) modality, surgical resection margins status, date of surgery, type of recurrence and date of first recurrence, as well as time of death and last follow-up.

### Statistical analysis

Primary endpoints of the study were disease-free survival (DFS) and overall survival (OS). DFS was measured from the date of surgery until the first documented tumor recurrence (distant or local) or death from any cause, whichever occurred first, or it was censored at the last follow-up. OS was defined as the time from surgery until time or death from any cause, or it was censored at the time of the last follow-up. Kaplan–Meier curves and log-rank tests were obtained to assess the DFS and OS probability and estimate the median OS and DFS, as well as to visualize key comparisons.

Baseline characteristics are reported as the median and interquartile range (IQR) for continuous variables and total number and proportions for categorical variables. Differences in baseline features by chemotherapy timing and RT modality were assessed using the Kruskal–Wallis test for continuous variables and Fisher exact test with Monte Carlo simulation for categorical variables.

Univariable associations with DFS and OS were assessed using Cox proportional hazards models. A multivariable Cox proportional hazard model was fitted for both OS and DFS, including variables that were significantly associated with OS or DFS in the univariable analysis as well as prespecified potential clinical confounders (resection margins, perioperative XRT and chemotherapy modality, tumor size and depth, posttreatment necrosis, age at diagnosis, and treating institution) using complete-case analysis. Nonlinear associations between tumor size and hazard were evaluated using Cox proportional hazards models for OS and DFS, adjusted for age, tumor depth, primary site, resection margin, and treating institution. Tumor size was modeled with a natural cubic spline (three degrees of freedom), and the results were visualized as hazard ratios (HR) relative to the median tumor size. Significance level was set to *P* < 0.05 for all analyses. Statistical analysis was performed in R (RRID: SCR_001905) using the packages survminer version 0.4.9 (RRID: SCR_021094; ref. 18), survival version 3.5-7 (RRID: SCR_021137; ref. 19), and coxphf version 1.13.4 (20).

## Results

### Demographics

A total of 248 patients diagnosed with SS between 2000 and 2024 at either the University of California San Francisco (UCSF), Stanford Health Care (SHC), or Beth Israel Deaconess Medical Center were reviewed and included in the study. Detailed patient demographics and baseline characteristics are reported in Table 1. Our cohort included 124 males (50%) and 124 females (50%). The median age at diagnosis was 35 years (IQR, 26–46 years). At the time of data collection and analysis, most patients were alive with no evidence of disease (*n* = 133, 53.6%), whereas 33.5% (*n* = 83) were deceased. Most patients were Caucasians (*n* = 156, 63%), followed by Asian (*n* = 31, 13%), Hispanic (*n* = 23, 9.3%), Black or African American (*n* = 20, 8.1%), and American Indian or Native Hawaiian/Pacific Islander (*n* = 2, 0.8% both). The median time since diagnosis was 5.2 years.

**Table 1.** Baseline patient characteristics.

Characteristic	Overall (*N* = 248)
Demographics	​
Gender	​
Male^a^	124 (50%)
Female	124 (50%)
Ethnicity	​
Caucasian	156 (63%)
Asian	31 (13%)
Hispanic	23 (9.3%)
Black or African American	20 (8.1%)
American Indian/Alaska Native	2 (0.8%)
Native Hawaiian/Pacific Islander	2 (0.8%)
Other	14 (5.6%)
Age at diagnosis, median, years (IQR)	35 (26–46)
Institution	​
UCSF	137 (55%)
SHC	86 (35%)
BIDMC	25 (10%)
Tumor characteristics	​
Tumor size max dimension, median, cm (IQR)	5.7 (3.5–8.7)
Liver nodules ≥1 cm at Dx	​
No	178 (72%)
Yes	4 (1.6%)
Unknown	66 (27%)
*SS18* fusion partner	​
*SSX1*	15 (6%)
*SSX2*	18 (7.2%)
Unknown	215 (86.6%)
Tumor depth	​
Deep	105 (42%)
Superficial	20 (8.1%)
Unknown	123 (50%)
Tumor site	​
Trunk/extremities/chest wall	225 (91%)
Other (visceral, retroperitoneal, and head and neck)	23 (9.3%)
Histology	​
Monophasic	144 (58%)
Biphasic	58 (23%)
Unknown	46 (19%)
T staging	​
T1 (<5 cm)	94 (38%)
T2 (5–10 cm)	96 (39%)
T3 (10–15 cm)	32 (13%)
T4 (≥15 cm)	15 (6%)
Missing	11 (4.4%)

Abbreviations: BIDMC, Beth Israel Deaconess Medical Center; Dx, diagnosis.

a *n* (%).

**Table 2.** Treatment received and outcomes.

Characteristic	Overall (*N* = 248)
Perioperative XRT	​
EBRT only (adjuvant or neoadjuvant)	130 (52%)
EBRT + IORT (adjuvant or neoadjuvant)	22 (8.9%)
IORT only	21 (8.5%)
No RT	75 (30.2%)
Adjuvant EBRT only	75 (30.2%)
Neoadjuvant EBRT only	49 (19.8%)
EBRT (adjuvant and neoadjuvant)	5 (2%)
Adjuvant EBRT only plus IORT	22 (8.9%)
Perioperative chemotherapy	​
Adjuvant only	58 (23%)
Neoadjuvant only	53 (21%)
Neoadjuvant and adjuvant	39 (16%)
No chemotherapy	98 (40%)
Type of recurrence	​
No recurrence^a^	150 (60%)
Distant	54 (22%)
Local	32 (13%)
Distant and local	12 (4.8%)
Neoadjuvant therapy	​
Neoadjuvant chemotherapy cycles number, median (IQR)	3 (1–5)
Perioperative XRT dose, Gy, median (IQR)	50 (45–55)
Perioperative XRT fractions, median (IQR)	25 (25–25)
Neoadjuvant XRT	​
None	156 (63%)
EBRT	54 (22%)
IORT	38 (15%)
Neoadjuvant chemotherapy	​
None	156 (63%)
AIM	81 (32.6%)
Ifosfamide	5 (2%)
AIM/IE	1 (0.4%)
Doxorubicin	1 (0.4%)
Epirubicin/ifosfamide	1 (0.4%)
IE	1 (0.4%)
Trabectedin	1 (0.4%)
VAC/AIM	1 (0.4%)
Surgery	​
Surgical resection margins	​
R0	159 (64%)
R1	36 (15%)
R2	13 (5.2%)
Missing	40 (16%)
Percent tumor necrosis at resection, median (IQR)	25 (0–50)
Adjuvant therapy	​
Adjuvant XRT	​
No	145 (58%)
EBRT	97 (39%)
EBRT and IORT	5 (2%)
Proton therapy	1 (0.4%)
Adjuvant chemotherapy	​
None	151 (60.8%)
AIM	87 (35.1%)
Ifosfamide	5 (2%)
Epirubicin/ifosfamide	2 (0.8%)
Doxorubicin	1 (0.4%)
Gemcitabine/docetaxel	1 (0.4%)
MAID	1 (0.4%)
Status at last follow-up	​
Alive, NED	134 (54%)
Alive with disease	45 (18.1%)
Deceased	69 (27.8%)

Abbreviations: AIM/IE, doxorubicin–idosfamide–mesna alternating with ifosfamide/etoposide; NED, no evidence of disease; VAC/AIM, doxorubicin–idosfamide–mesna alternating with vincristine–dactinomycin–cyclophosphamide.

a *n* (%).

### Tumor characteristics

The median tumor size at diagnosis was 5.7 cm (range, 3.5–8.7 cm), with most patients being classified as stage T2 (*n* = 96, 39%) or T1 (*n* = 94, 38%). Most patients had tumors located in the trunk, chest wall, or extremities (*n* = 225, 91%), with less than 10% in locations such as the head and neck, retroperitoneum, or a visceral location (*n* = 23, 9.3%). Most patients had a monophasic SS histology (*n* = 144, 58%). Most patients had a deep location with invasion of the fascia (*n* = 105, 42.9%).

### Treatment

Neoadjuvant chemotherapy was administered to 92 patients (37.1%), with the most common regimen being doxorubicin–ifosfamide–mesna (AIM), given to 86 patients (34.7%). Of all the patients that received neoadjuvant chemotherapy, 39 patients (16%) also received adjuvant chemotherapy. The median number of neoadjuvant chemotherapy cycles was 3 (IQR, 1–5; Table 2).

Overall, RT was administered to 173 (69.7%) patients. EBRT was administered to 54 patients (22%) before surgery, whereas 97 patients (39%) received EBRT in the adjuvant setting, and of these, 75 patients received adjuvant EBRT only (*n* = 22, 8.9%). Of the 54 patients that received neoadjuvant EBRT, 39 received concurrent neoadjuvant chemotherapy. IORT was administered to 43 patients (17.3%), either as the sole RT modality (*n* = 21, 8.5%) or in combination with adjuvant EBRT (*n* = 22, 8.9%). The median perioperative XRT dose was 50 Gy (IQR, 45–55 Gy) administered in a median number of 25 fractions (IQR, 25–25). Most patients had R0 resection margins at surgery (159, 64%), with median percent of tumor necrosis on the resection specimen of 25% (IQR, 0%–50%). Adjuvant chemotherapy was administered to 97 patients (39.1%), with AIM being the most common regimen (*n* = 87, 35.1%). Adjuvant XRT was given to 102 patients (41.1%), including both patients with R0 (*n* = 52/102, 50.9%) and R1 or R2 (*n* = 30/102, 29.4%) resection margins. Most patients did not have disease recurrence at the time of the last follow-up (*n* = 134, 54%), with the most common recurrence being metastatic (*n* = 54, 21.8%; Table 2).

### DFS

The median DFS for the whole cohort was 110.3 months [95% confidence interval (CI), 57.6–not reached], whereas the 2- and 5-year DFS rates were 71.9% and 55.9%, respectively. Patients with primary site involving the trunk, chest wall, or extremities had better DFS risk compared with patients with SS involving other sites (HR, 0.46; 95% CI, 0.27–0.77; *P* = 0.003; Table 3). Tumor size continuously and positively correlated with worse DFS (HR, 1.12; 95% CI, 1.09–1.16; *P* < 0.001; Supplementary Fig. S1A), as well as age (HR, 1.01; 95% CI, 1.00–1.02; *P* = 0.047) and the percent of tumor necrosis on the resection specimen (HR, 1.01; 95% CI, 1–1.02; *P* = 0.001).

**Table 3.** Cox univariable analysis for DFS and OS associations.

Variable	Level	DFS HR (95% CI)	DFS *P* value (level)	DFS *P* value (global)	OS HR (95% CI)	OS *P* value (level)	OS *P* value (global)
Age (years)	Per unit increase	1.01 (1–1.02)	**0.047**	0.050	1.02 (1–1.03)	**0.030**	**0.032**
Gender	Male	0.91 (0.63–1.31)	0.612	0.612	1.08 (0.67–1.73)	0.758	0.758
Ethnicity	Asian	0.54 (0.07–4.12)	0.550	0.617	0.14 (0.02–1.18)	0.071	0.055
	Black or African American	0.52 (0.06–4.26)	0.544	​	0.22 (0.03–1.90)	0.168	​
	Caucasian	0.67 (0.09–4.85)	0.694	​	0.25 (0.03–1.85)	0.175	​
	Hispanic	0.50 (0.06–3.94)	0.509	​	0.09 (0.01–0.91)	**0.041**	​
	Native Hawaiian/Pacific Islander	2.68 (0.24–29.72)	0.421	​	1.85 (0.17–20.46)	0.615	​
	Other	0.56 (0.06–4.77)	0.593	​	0.13 (0.01–1.43)	0.096	​
Tumor size (cm)	Per unit increase	1.12 (1.09–1.16)	**<0.001**	**<0.001**	1.21 (1.16–1.27)	**<0.001**	**<0.001**
Tumor depth	Missing	0.91 (0.62–1.33)	0.618	0.693	0.69 (0.42–1.13)	0.144	0.341
	Superficial	0.74 (0.35–1.56)	0.432	​	0.77 (0.32–1.84)	0.556	​
Primary site	Trunk/chest wall/extremities	0.46 (0.27–0.77)	**0.003**	**0.007**	0.45 (0.24–0.84)	**0.012**	**0.021**
Resection margins	R0	0.74 (0.46–1.19)	0.212	0.392	0.58 (0.33–1.02)	0.059	**0.027**
	R1	0.56 (0.28–1.12)	0.103	​	0.27 (0.09–0.81)	**0.019**	​
	R2	0.87 (0.37–2.02)	0.741	​	1.13 (0.47–2.72)	0.793	​
Institution	SHC	1 (0.47–2.15)	0.990	0.983	1.04 (0.31–3.51)	0.954	0.427
	UCSF	0.97 (0.46–2.02)	0.933	​	1.44 (0.45–4.64)	0.543	​
Percent necrosis	Per unit increase	1.01 (1–1.02)	**0.001**	**0.002**	1.01 (1–1.02)	**0.006**	**0.008**
Liver nodules ≥1 cm at Dx	No	0.61 (0.41–0.90)	**0.012**	**0.040**	0.82 (0.49–1.37)	0.451	0.670
	Yes	0.39 (0.05–2.87)	0.358	​	1.50 (0.20–11.26)	0.692	​
Perioperative chemotherapy	Adjuvant only	2.06 (1.30–3.26)	**0.002**	**0.020**	2.03 (1.13–3.65)	**0.018**	0.105
	Both	1.63 (0.92–2.92)	0.097	​	1.77 (0.83–3.76)	0.138	​
	Neoadjuvant only	1.41 (0.84–2.37)	0.191	​	1.54 (0.78–3.03)	0.212	​
Perioperative RT modality	EBRT only	1.28 (0.85–1.92)	0.233	**0.002**	1.01 (0.61–1.67)	0.963	**<0.001**
	EBRT + IORT	0.62 (0.29–1.34)	0.223	​	0.40 (0.14–1.16)	0.092	​
	IORT only	0.30 (0.11–0.83)	**0.021**	​	0.09 (0.01–0.66)	**0.018**	​
Neoadjuvant chemotherapy regimen	AIM	0.94 (0.62–1.42)	0.777	**0.007**	1.09 (0.64–1.85)	0.743	0.439
	Not AIM	3.96 (1.89–8.28)	**<0.001**	​	2.09 (0.75–5.81)	0.160	​
Adjuvant chemotherapy regimen	AIM	1.66 (1.13–2.44)	**0.009**	**0.023**	1.69 (1.04–2.75)	**0.035**	0.104
	Not AIM	1.84 (0.84–4.02)	0.128	​	1.57 (0.56–4.42)	0.393	​

The bold values indicate statistical significance value of *P* < 0.05.

Abbreviation: Dx, diagnosis.

**Figure 1.**
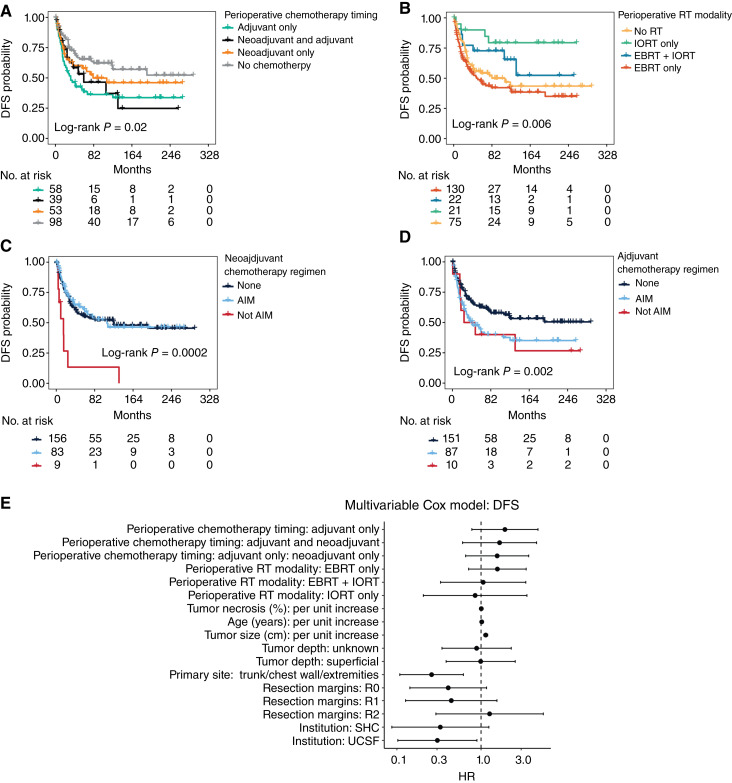
DFS associations. **A,** Kaplan–Meier curve showing DFS probability based on perioperative chemotherapy timing. **B,** Kaplan–Meier curve showing DFS probability based on the modality of perioperative XRT. **C,** Kaplan–Meier curve showing DFS probability for patients receiving neoadjuvant AIM, those receiving neoadjuvant chemotherapy regimens different than AIM, and those receiving no neoadjuvant chemotherapy. **D,** Kaplan–Meier curve showing DFS probability for patients receiving adjuvant AIM, those receiving adjuvant chemotherapy regimens different than AIM, and those receiving no adjuvant chemotherapy. **E,** Forest plot showing results of multivariable Cox proportional hazard analysis for DFS.

Patients receiving adjuvant chemotherapy only had worse DFS in the univariate analysis compared with those that did not receive any perioperative chemotherapy (HR, 2.06; 95% CI, 1.30–3.26; *P* = 0.002; Fig. 1A), whereas there was no difference in DFS risk based on perioperative chemotherapy modality in the multivariable Cox analysis (*P* = 0.549; Table 4). This is likely explained by differences in baseline patient and tumor characteristics (Supplementary Table S1) as patients that did not receive perioperative chemotherapy tended to have smaller tumors with a median tumor size of 3.2 cm (IQR, 2–5.1 cm) compared with those that received adjuvant (median size: 6.1 cm, IQR: 4.7–9.6 cm), neoadjuvant (median size: 7.4 cm, IQR: 5.2–11.3 cm), or both adjuvant and neoadjuvant chemotherapy (median size: 8.2 cm, IQR: 6.1–10.7 cm, *P* < 0.001).

**Table 4.** Multivariable Cox regression (DFS and OS from surgery).

Variable	Level	DFS HR (95% CI)	DFS *P* value (level)	DFS *P* value (global)	OS HR (95% CI)	OS *P* value (level)	OS *P* value (global)
Perioperative chemotherapy	Neoadjuvant only	1.56 (0.66–3.69)	0.316	0.549	1.50 (0.40–5.58)	0.548	0.414
	Adjuvant only	1.92 (0.78–4.75)	0.157	​	2.14 (0.68–6.75)	0.195	​
	Both	1.66 (0.60–4.57)	0.326	​	2.54 (0.66–9.73)	0.175	​
Perioperative RT modality	EBRT only	1.57 (0.71–3.44)	0.265	0.572	1.05 (0.40–2.74)	0.928	0.401
	IORT only	0.85 (0.21–3.51)	0.824	​	0.26 (0.03–2.49)	0.244	​
	EBRT + IORT	1.06 (0.33–3.40)	0.921	​	0.44 (0.08–2.44)	0.350	​
Percent necrosis	Per unit increase	1.01 (1–1.02)	0.168	0.168	1.01 (1–1.03)	**0.048**	**0.049**
Age (years)	Per unit increase	1.02 (1–1.04)	0.081	0.083	1.03 (1–1.06)	0.074	0.078
Tumor size (cm)	Per unit increase	1.14 (1.08–1.20)	**<0.001**	**<0.001**	1.29 (1.18–1.40)	**<0.001**	**<0.001**
Tumor depth	Unknown	0.89 (0.34–2.29)	0.803	0.969	0.80 (0.17–3.83)	0.783	0.942
	Superficial	0.99 (0.38–2.55)	0.984	​	0.86 (0.22–3.32)	0.822	​
Primary site	Trunk/chest wall/extremities	0.26 (0.11–0.62)	**0.002**	**0.006**	0.61 (0.21–1.79)	0.366	0.379
Resection margins	R0	0.41 (0.14–1.16)	0.093	0.199	0.10 (0.02–0.43)	**0.002**	**0.004**
	R1	0.44 (0.13–1.55)	0.202	​	0.04 (0–0.41)	**0.008**	​
	R2	1.26 (0.29–5.49)	0.755	​	0.67 (0.13–3.42)	0.632	​
Institution	Stanford	0.33 (0.09–1.24)	0.100	0.120	0.50 (0.04–6.29)	0.594	0.847
	UCSF	0.30 (0.10–0.89)	**0.031**	​	0.77 (0.07–8.34)	0.833	​

The bold values indicate statistical significance value of *P* < 0.05.

Also, in the univariate analysis, patients receiving IORT had better DFS risk (HR, 0.30; 95% CI, 0.11–0.83; *P* = 0.02) than those receiving EBRT only or EBRT plus IORT (Fig. 1B), though there were no significant associations between DFS and perioperative RT modality in the multivariate analysis (*P* = 0.572; Table 4). Again, this is likely explained by variability in baseline patient characteristics, as patients that received IORT tended to have smaller tumors (median tumor size 4 cm, IQR: 2.1–5 cm) compared with those that received EBRT (median size: 6.6 cm, IQR: 4.5–9.3 cm) or EBRT + IORT (median size: 5.8 cm, IQR: 3.9–7.9 cm, *P* = 0.005). Also, notably, there was a significant institution bias in the administration of IORT where this was given as a standalone RT modality only at the UCSF or as a combination with EBRT only at the UCSF and SHC (*P* < 0.001; Supplementary Table S2). In the univariate analysis, administration of a neoadjuvant chemotherapy regimen different than AIM correlated with worse DFS (HR, 3.96; 95% CI, 1.89–8.28; *P* < 0.001) as opposed to receiving neoadjuvant AIM or no neoadjuvant chemotherapy (Fig. 1C), whereas patients that received adjuvant AIM had worse DFS (HR, 1.66; 95% CI, 1.13–2.44; *P* = 0.09) compared with those receiving a chemotherapy regimen different than AIM or no chemotherapy (Fig. 1D). However, given the small number of patients receiving chemotherapy regimens different than AIM, either in the neoadjuvant or adjuvant setting, as well as differences in baseline patient characteristics based on perioperative chemotherapy modality (Supplementary Table S1), these findings should be considered exploratory in nature. In the Cox proportional hazards multivariable analysis (Fig. 1E; Table 4), tumor size (HR, 1.13; 95% CI, 1.08–1.17; *P* < 0.001) correlated with worse DFS, whereas a primary tumor site involving the chest wall, extremities, or trunk was associated with better DFS risk (HR, 0.26; 95% CI, 0.11–0.62; *P* < 0.006). Lastly, we performed an exploratory subgroup analysis considering only patients with tumors of size ≥10 cm and found no differences in DFS based on combined perioperative RT and chemotherapy modality either in the univariable analysis (Supplementary Fig. S1B; Supplementary Table S3) or in the multivariable analysis (Supplementary Table S4).

### OS

**Figure 2.**
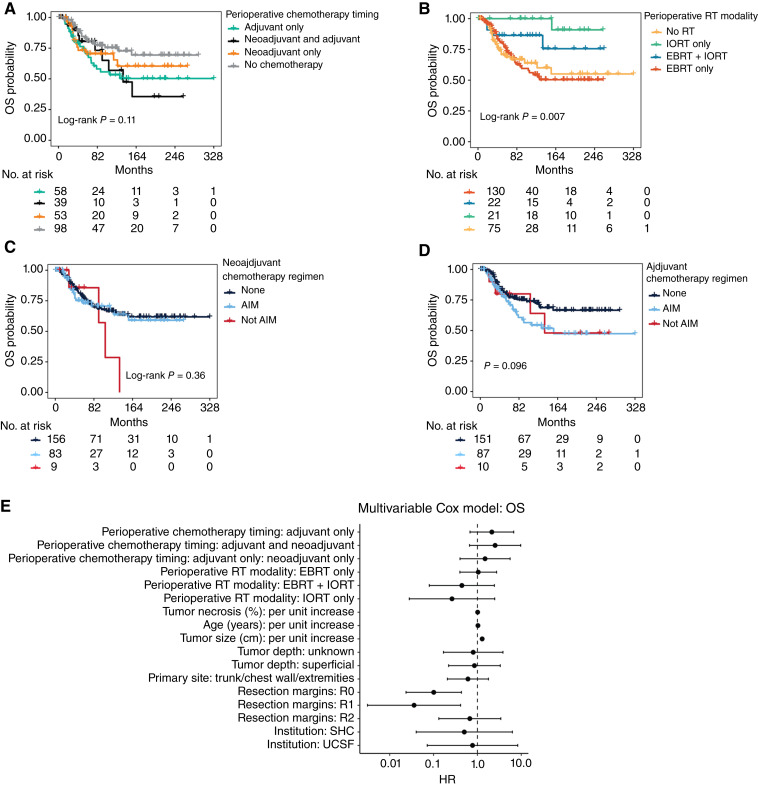
OS associations. **A,** Kaplan–Meier curve showing OS probability based on perioperative chemotherapy timing. **B,** Kaplan–Meier curve showing OS probability based on the modality of perioperative XRT. **C,** Kaplan–Meier curve showing OS probability for patients receiving neoadjuvant AIM, those receiving neoadjuvant chemotherapy regimens different than AIM, and those receiving no neoadjuvant chemotherapy. **D,** Kaplan–Meier curve showing OS probability for patients receiving adjuvant AIM, those receiving adjuvant chemotherapy regimens different than AIM, and those receiving no adjuvant chemotherapy. **E,** Forest plot showing results of multivariable Cox proportional hazard analysis for OS.

The median OS for the whole cohort was not reached (95% CI, not reached–not reached), whereas the 2- and 5-year OS rates were 94.7% and 77.1%, respectively. A primary tumor location involving the chest wall, trunk, or extremities correlated with improved OS (HR, 0.45; 95% CI, 0.24–0.84; *P* = 0.012), as well as R1 resection margins (HR, 0.27; 95% CI, 0.09–0.81; *P* = 0.019; Table 3). Patient age (HR, 1.02; 95% CI, 1–1.03; *P* = 0.03), tumor size (HR, 1.21; 95% CI, 1.16–1.27; *P* < 0.001; Supplementary Fig. S1C), and percent tumor necrosis on the resection specimen (HR, 1.01; 95% CI, 1–1.02; *P* = 0.006) all correlated with worse OS risk in the univariate analysis. Similarly to what was observed for the DFS analysis, in the univariate OS analysis also we observed worse OS risk for patients that received only adjuvant chemotherapy as opposed to no chemotherapy (HR, 2.03; 95% CI, 1.13–3.65; *P* = 0.018; Fig. 2A; Table 3), though this association was not present in the Cox multivariable analysis (*P* = 0.548; Table 4). Also, in the univariate analysis, patients receiving only IORT as perioperative RT modality showed improved OS (HR, 0.09; 95% CI, 0.01–0.66; *P* = 0.018; Fig. 2B; Table 3), though this was not true in the multivariable analysis (*P* = 0.244; Table 4). These findings are analogous to what observed for DFS and could again be explained by differences in baseline patient characteristics and bias related to the treating institution (Supplementary Tables S1 and S2). Also, we observed no differences in OS based on chemotherapy regimen administered, comparing AIM with regimens different than AIM or with no chemotherapy, either in the neoadjuvant (*P* = 0.493, Fig. 2C) or adjuvant setting (*P* = 0.104, Fig. 2D). In the multivariable cox analysis (Fig. 2E; Table 4), the only variables associated with worse OS risk were percent tumor necrosis (HR, 1.01; 95% CI, 1–1.03; *P* = 0.048) and tumor size (HR, 1.29; 95% CI, 1.18–1.40; *P* < 0.001), whereas R0 (HR, 0.10; 95% CI, 0.02–0.43; *P* = 0.002) or R1 (HR, 0.04; 95% CI, 0–0.41; *P* = 0.008) surgical resection margins correlated with better OS. Considering only patients with tumor size ≥10 cm, we found that those receiving adjuvant or neoadjuvant chemotherapy together with perioperative RT had longer OS (median OS, 8.9; 95% CI, 3.8–not reached) compared with those receiving perioperative chemotherapy only (median OS, 3.1; 95% CI, 2.5–not reached; Log-Rank *P* = 0.04; Supplementary Fig. S1D). Also, in the cox multivariable analysis, OS was worse for patients with tumor size ≥10 cm that received only either adjuvant or neoadjuvant chemotherapy (HR, 5.29; 95% CI, 1.61–17.39; *P* = 0.006) as opposed to those receiving both chemotherapy and RT (Supplementary Table S4).

## Discussion

Here, we report on 248 patients with localized SS treated at three different sarcoma centers in the United States, representing one of the largest independent cohorts described to date of patients with localized SS. The demographic features of patients in our cohort matched what is described in the literature, with a median age at diagnosis of 35 years and with no significant difference in incidence between men and women (21). In our cohort, median OS was not reached with a median follow-up time of 5.2 years. This is similar to what was observed in the Surveillance, Epidemiology, and End Results cohort for patients with localized SS, in which the median OS for patients with localized disease was 366 months (21), but significantly longer than what reported by Robertson-Smith and colleagues (22), in which the median OS for a cohort of 94 patients with localized SS treated in the United Kingdom was 83 months, with a mean follow-up time of 70 months. These cross-study differences in survival should be interpreted cautiously given the retrospective nature of both studies and differences in cohort composition, perioperative treatment selection, follow-up duration, local referral practices, and stage at presentation.

Our study confirmed the prognostic role of several clinicopathologic and treatment-related features that correlated with DFS and OS. Specifically, tumor size was a strong and independent continuous predictor of both DFS and OS. This is consistent with multiple prior studies showing worse outcomes for patients with larger tumors. Notably, in a series of adult patients with localized SS reported on by Sacchetti and colleagues (23), tumor size >5 cm correlated with significantly reduced OS (2.8 HR for death linked to cancer). Also, Vining and colleagues (24) showed that patients with stage T3 tumors (10–15 cm in largest diameter) tended to have worse outcomes and were the ones to benefit the most from adjuvant chemotherapy. Similarly, in pediatric patients with localized SS, tumor size greater than 5 cm correlated with worse OS (25, 26). This is likely related to increased metastatic potential and more difficult local control associated with larger tumor size. Interestingly, although we observed no association with DFS or OS for either perioperative chemotherapy or RT in the whole cohort, a subgroup analysis of patients with tumors larger than 10 cm showed an OS benefit for the combination of adjuvant or neoadjuvant chemotherapy with perioperative RT, which seems to be consistent with prior data indicating that a more aggressive multimodal perioperative management might be beneficial for patients with high-risk features (17). However, the small sample size of our subgroup analysis warrants cautious interpretation of these results with need for validation in prospective interventional studies.

In our cohort, achieving negative (R0) or microscopically positive (R1) surgical resection margins correlated with improved OS in the Cox multivariable analysis. This is again consistent with prior reports in both adult and pediatric patients (23, 27) and underscores the importance of local disease control in patients with localized SS. Also, we found that the percent of tumor necrosis on the resection specimen independently correlated with worse OS. This is consistent with findings from Trassard and colleagues (8) and might be a proxy for higher grade and a more aggressive underlying biology, rather than representing treatment effect for those patients that received neoadjuvant treatment.

Regarding local therapy, our data showed improved OS and DFS for patients receiving IORT as the sole RT modality, though these findings only held true in the univariate analysis but not in the multivariate analysis. These findings were likely confounded by selection bias, as patients offered IORT had smaller tumors, which is in line with prior literature in which patients offered IORT usually tend to have a more favorable and surgically accessible tumor location which might lead to improved local control and overall outcomes (28). Also, IORT was mainly administered at the UCSF which introduces bias related to the treating institution. Nevertheless, a direct comparison of IORT and EBRT in patients with SS is lacking, and our results, though limited, might encourage further prospective study of this XRT modality in a direct comparison against EBRT.

Regarding perioperative systemic therapy, our analysis does not support conclusions about the comparative efficacy of specific chemotherapy regimens, given the small number of patients receiving perioperative chemotherapy regimens different than AIM. Also, although patients treated with adjuvant chemotherapy alone had worse OS and DFS in the univariate analysis, these associations were not retained after multivariable adjustment for confounders and clinicopathologic factors. Further analysis indeed confirmed that patients that did not receive chemotherapy tended to have smaller tumors, whereas tumor size was instead a strong continuous predictor of both OS and DFS. These findings are consistent with confounding by indication given the retrospective nature of our study. Additionally, our findings of improved OS among patients with tumor size ≥10 cm who received combined perioperative chemotherapy and RT, as opposed to chemotherapy alone, is aligned with prior clinical studies in patients with localized SS and other STS histologies. Prior retrospective studies in patients with localized SS showed benefit from perioperative ifosfamide-based chemotherapy regimens in patients with high-risk primary SS of the extremities (10). Also, broader retrospective series including patients with high-risk STS of the extremities indicated benefit from high-dose perioperative chemotherapy, or combined perioperative chemotherapy and RT, in patients with larger tumors and high-risk features (10, 11, 29, 30). At the same time, other large retrospective SS studies have not demonstrated a clear survival benefit for perioperative chemotherapy after adjustment for clinicopathologic features (31), underscoring the ongoing uncertainty and the need for prospective validation.

### Conclusion

Collectively, our results support the need for improved risk stratification and more precise use of currently available perioperative treatments in patients with localized SS, while also highlighting the need for new therapeutic approaches to improve DFS and OS. In our cohort, outcomes were most strongly associated with established clinicopathologic factors, including tumor size and resection margin status, and patients with higher-risk features seemed to derive the greatest potential benefit from intensified multimodal perioperative management. At the same time, our analyses cannot support definitive conclusions about the benefit of specific chemotherapy regimens, and treatment-effect estimates should be interpreted cautiously given the retrospective design and residual confounding by indication. Our data also suggest that IORT may warrant further prospective evaluation as part of perioperative RT strategies aimed at improving local control in selected patients. At the same time, our results show improved outcomes for patients with smaller tumors and for those with R0 resection, suggesting the need to study deescalating treatment modalities, as it has been demonstrated in the pediatric population in which surgery with or without XRT achieves good outcomes for patients with low-risk localized SS (25, 32).

It must be noted that our study presents significant limitations due to its retrospective nature, including confounding by indication in treatment selection. Also, the multi-institutional nature of our study introduces bias due to variability in staging and response assessment across different sarcoma centers. The relatively small numbers in certain subgroups can limit statistical power and does preclude definitive conclusions. Additionally, data on chemotherapy regimen dosing and treatment‐related toxicities were not uniformly captured, which may influence accurate interpretation of efficacy of each systemic regimen. Lastly, certain patient-related features, as *SS18* fusion status, were not available for all patients which precluded our ability to assess their prognostic value. Further prospective studies are needed to further refine the optimal perioperative chemotherapy regimen for patients with localized SS, as it pertains to number of cycles and possibility of dose reductions. Ultimately, a risk-adapted management approach which stratifies patients by tumor size, margin status, and metastatic risk, will be critical to maximize cure rates while minimizing treatment-related morbidity in patients with localized SS.

## Supplementary Material

Supplementary Figure S1. Supplementary Figure S1 showing associations between tumor size and outcomes.

Supplementary Table S1. Supplementary Table S1. Baseline features by perioperative chemotherapy timing.

Supplementary Table S2. Supplementary Table S2. Baseline features by perioperative radiotherapy modality.

Supplementary Table S3. Supplementary Table S3. Tumor size ≥10 cm subgroup Cox univariable models for DFS and OS from surgery.

Supplementary Table S4. Supplementary Table S4. Tumor size ≥10 cm subgroup Cox multivariable models for DFS and OS from surgery.

## Data Availability

Primary data are available from the corresponding author upon reasonable request.
