# Supplementary material for: A Multicenter Retrospective Outcomes Analysis of Patients with Localized Synovial Sarcoma
Source: Cancer Res Commun. 2026 Jun 3;6(6):1295–304. doi: 10.1158/2767-9764.CRC-25-0652 (PMC13231045; doi:10.1158/2767-9764.CRC-25-0652)
Supplement: Supplementary Table S1. — Baseline features by perioperative chemotherapy timing. [file crc-25-0652_supplementary_table_s1.suppst1.docx]

# **Supplementary Table S1. Baseline features by perioperative chemotherapy timing.**

| **Characteristic** | **N** | **No Chemotherapy**, N = 98^1^ | **Neoadjuvant Only**, N = 53^1^ | **Adjuvant Only**, N = 58^1^ | **Neoadjuvant and Adjuvant**, N = 39^1^ | **P-value**^2^ |
| --- | --- | --- | --- | --- | --- | --- |
| Age at diagnosis (yrs) | 248 | 36 (27, 54) | 35 (26, 42) | 39 (23, 49) | 32 (26, 40) | 0.2 |
| Tumor size (cm) | 237 | 3.2 (2.0, 5.1) | 7.4 (5.2, 11.3) | 6.1 (4.7, 9.6) | 8.2 (6.1, 10.7) | **<0.001** |
| Unknown |  | 7 | 1 | 3 | 0 |  |
| T Staging | 248 |  |  |  |  | **<0.001** |
| Missing |  | 7 (7.1%) | 1 (1.9%) | 3 (5.2%) | 0 (0%) |  |
| T1 (<5 cm) |  | 63 (64%) | 11 (21%) | 15 (26%) | 5 (13%) |  |
| T2 (5-10 cm) |  | 23 (23%) | 25 (47%) | 29 (50%) | 19 (49%) |  |
| T3 (10-15 cm) |  | 3 (3.1%) | 11 (21%) | 7 (12%) | 11 (28%) |  |
| T4 (≥ 15 cm) |  | 2 (2.0%) | 5 (9.4%) | 4 (6.9%) | 4 (10%) |  |
| Tumor Site | 248 |  |  |  |  | 0.052 |
| Other (visceral, retroperitoneal, head and neck) |  | 9 (9.2%) | 5 (9.4%) | 9 (16%) | 0 (0%) |  |
| Trunk/extremities/chest wall |  | 89 (91%) | 48 (91%) | 49 (84%) | 39 (100%) |  |
| Tumor Depth | 248 |  |  |  |  | **0.005** |
| Deep |  | 31 (32%) | 30 (57%) | 28 (48%) | 16 (41%) |  |
| Unknown |  | 57 (58%) | 16 (30%) | 29 (50%) | 21 (54%) |  |
| Superficial |  | 10 (10%) | 7 (13%) | 1 (1.7%) | 2 (5.1%) |  |
| Resection Margins (R0/R1/R2) | 248 |  |  |  |  | **0.027** |
| Missing |  | 21 (21%) | 9 (17%) | 9 (16%) | 1 (2.6%) |  |
| R0 |  | 56 (57%) | 32 (60%) | 39 (67%) | 32 (82%) |  |
| R1 |  | 16 (16%) | 11 (21%) | 4 (6.9%) | 5 (13%) |  |
| R2 |  | 5 (5.1%) | 1 (1.9%) | 6 (10%) | 1 (2.6%) |  |
| Institution | 248 |  |  |  |  | **<0.001** |
| BIDMC |  | 16 (16%) | 6 (11%) | 0 (0%) | 3 (7.7%) |  |
| Stanford |  | 38 (39%) | 9 (17%) | 23 (40%) | 16 (41%) |  |
| UCSF |  | 44 (45%) | 38 (72%) | 35 (60%) | 20 (51%) |  |
| ^1^Median (IQR); n (%) | | | | | | |
| ^2^Kruskal-Wallis rank sum test; Fisher's Exact Test for Count Data with simulated p-value  (based on 20000 replicates) | | | | | | |
