# Supplementary material for: A Multicenter Retrospective Outcomes Analysis of Patients with Localized Synovial Sarcoma
Source: Cancer Res Commun. 2026 Jun 3;6(6):1295–304. doi: 10.1158/2767-9764.CRC-25-0652 (PMC13231045; doi:10.1158/2767-9764.CRC-25-0652)
Supplement: Supplementary Table S2. — Baseline features by perioperative radiotherapy modality. [file crc-25-0652_supplementary_table_s2.suppst2.docx]

# **Supplementary Table S2. Baseline features by perioperative radiotherapy modality.**

| **Characteristic** | **N** | **No RT**, N = 75^1^ | **EBRT Only**, N = 130^1^ | **IORT Only**, N = 21^1^ | **EBRT+IORT**, N = 22^1^ | **P-value**^2^ |
| --- | --- | --- | --- | --- | --- | --- |
| Age at diagnosis (yrs) | 248 | 36 (24, 48) | 35 (27, 45) | 27 (12, 37) | 35 (30, 42) | 0.2 |
| Tumor size (cm) | 237 | 5.0 (2.7, 9.1) | 6.6 (4.5, 9.3) | 4.0 (2.1, 5.0) | 5.8 (3.9, 7.9) | **0.005** |
| Unknown |  | 5 | 6 | 0 | 0 |  |
| T Staging | 248 |  |  |  |  | **0.011** |
| Missing |  | 5 (6.7%) | 6 (4.6%) | 0 (0%) | 0 (0%) |  |
| T1 (<5 cm) |  | 34 (45%) | 37 (28%) | 15 (71%) | 8 (36%) |  |
| T2 (5-10 cm) |  | 19 (25%) | 61 (47%) | 5 (24%) | 11 (50%) |  |
| T3 (10-15 cm) |  | 11 (15%) | 18 (14%) | 0 (0%) | 3 (14%) |  |
| T4 (≥ 15 cm) |  | 6 (8.0%) | 8 (6.2%) | 1 (4.8%) | 0 (0%) |  |
| Tumor Site | 248 |  |  |  |  | 0.076 |
| Other (visceral, retroperitoneal, head and neck) |  | 4 (5.3%) | 18 (14%) | 0 (0%) | 1 (4.5%) |  |
| Trunk/extremities/chest wall |  | 71 (95%) | 112 (86%) | 21 (100%) | 21 (95%) |  |
| Tumor Depth | 248 |  |  |  |  | 0.2 |
| Deep |  | 27 (36%) | 56 (43%) | 11 (52%) | 11 (50%) |  |
| Unknown |  | 43 (57%) | 65 (50%) | 6 (29%) | 9 (41%) |  |
| Superficial |  | 5 (6.7%) | 9 (6.9%) | 4 (19%) | 2 (9.1%) |  |
| Resection Margins (R0/R1/R2) | 248 |  |  |  |  | 0.2 |
| Missing |  | 13 (17%) | 22 (17%) | 3 (14%) | 2 (9.1%) |  |
| R0 |  | 55 (73%) | 75 (58%) | 14 (67%) | 15 (68%) |  |
| R1 |  | 6 (8.0%) | 25 (19%) | 2 (9.5%) | 3 (14%) |  |
| R2 |  | 1 (1.3%) | 8 (6.2%) | 2 (9.5%) | 2 (9.1%) |  |
| Institution | 248 |  |  |  |  | **<0.001** |
| BIDMC |  | 4 (5.3%) | 21 (16%) | 0 (0%) | 0 (0%) |  |
| Stanford |  | 31 (41%) | 50 (38%) | 0 (0%) | 5 (23%) |  |
| UCSF |  | 40 (53%) | 59 (45%) | 21 (100%) | 17 (77%) |  |
| ^1^Median (IQR); n (%) | | | | | | |
| ^2^Kruskal-Wallis rank sum test; Fisher's Exact Test for Count Data with simulated p-value  (based on 20000 replicates) | | | | | | |
