# Supplementary material for: A Multicenter Retrospective Outcomes Analysis of Patients with Localized Synovial Sarcoma
Source: Cancer Res Commun. 2026 Jun 3;6(6):1295–304. doi: 10.1158/2767-9764.CRC-25-0652 (PMC13231045; doi:10.1158/2767-9764.CRC-25-0652)
Supplement: Supplementary Table S3. — Tumor size ≥10 cm subgroup Cox univariable models for DFS and OS from surgery. [file crc-25-0652_supplementary_table_s3.suppst3.docx]

# **Supplementary Table S3. Tumor size ≥10 cm subgroup Cox univariable models for DFS and OS from surgery.**

| **Variable** | **Level** | **DFS HR (95% CI)** | **DFS *P*-value (level)** | **DFS *P*-value (global)** | **OS HR (95% CI)** | **OS *P*-value (level)** | | **OS *P*-value (global)** |
| --- | --- | --- | --- | --- | --- | --- | --- | --- |
| Perioperative Treatment | No adjuvant or neoadjuvant chemotherapy, No perioperative RT | 1.37 (0.31–6.12) | 0.682 | 0.299 | 3.05 (0.61–15.28) | | 0.176 | 0.071 |
|  | Adjuvant or neoadjuvant chemotherapy, no perioperative RT | 1.13 (0.53–2.40) | 0.756 |  | 2.68 (1.05–6.87) | | **0.039** |  |
|  | Perioperative RT only | 6.28 (1.29–30.43) | **0.023** |  | 6.82 (1.32–35.26) | | **0.022** |  |
| Age (years) | Per unit increase | 1.03 (1.00–1.06) | **0.045** | **0.045** | 1.03 (1.00–1.06) | | 0.054 | 0.051 |
| Tumor depth | Unknown | 0.93 (0.46–1.91) | 0.849 | 0.746 | 0.66 (0.28–1.57) | | 0.348 | 0.630 |
|  | superficial | 0.58 (0.13–2.57) | 0.474 |  | 0.72 (0.16–3.31) | | 0.673 |  |
| Tumor site | Trunk/extremities/chest wall | 0.44 (0.18–1.09) | 0.077 | 0.104 | 0.52 (0.19–1.45) | | 0.212 | 0.238 |
| Institution (Stanford/BIDMC vs UCSF) | Stanford/BIDMC | 2.67 (1.23–5.82) | **0.013** | **0.015** | 1.15 (0.46–2.88) | | 0.760 | 0.762 |
