# Supplementary material for: A Multicenter Retrospective Outcomes Analysis of Patients with Localized Synovial Sarcoma
Source: Cancer Res Commun. 2026 Jun 3;6(6):1295–304. doi: 10.1158/2767-9764.CRC-25-0652 (PMC13231045; doi:10.1158/2767-9764.CRC-25-0652)
Supplement: Supplementary Table S4. — Tumor size ≥10 cm subgroup Cox multivariable models for DFS and OS from surgery. [file crc-25-0652_supplementary_table_s4.suppst4.docx]

# **Supplementary Table S4. Tumor size ≥10 cm subgroup Cox multivariable models for DFS and OS from surgery.**

| **Variable** | **Level** | **DFS HR  (95% CI)** | **DFS *P*-value (level)** | **DFS *P*-value (global)** | **OS HR  (95% CI)** | **OS *P*-value (level)** | **OS *P*-value (global)** |
| --- | --- | --- | --- | --- | --- | --- | --- |
| Perioperative Treatment | No adjuvant or neoadjuvant chemotherapy, No perioperative RT | 0.38 (0.04–3.86) | 0.412 | 0.214 | 4.54 (0.57–36.08) | 0.153 | **0.046** |
|  | Adjuvant or neoadjuvant chemotherapy, no perioperative RT | 1.53 (0.65–3.63) | 0.334 |  | 5.29 (1.61–17.39) | **0.006** |  |
|  | Perioperative RT only | 3.04 (0.32–28.46) | 0.330 |  | 9.19 (0.76–110.85) | 0.081 |  |
| Age (years) | Per unit increase | 1.04 (1.00–1.07) | **0.028** | **0.031** | 1.03 (1.00–1.08) | 0.080 | 0.081 |
| Tumor depth | Unknown | 0.51 (0.20–1.27) | 0.148 | 0.232 | 0.58 (0.18–1.92) | 0.377 | 0.157 |
|  | superficial | 0.34 (0.06–1.92) | 0.223 |  | 0.16 (0.02–1.20) | 0.074 |  |
| Tumor site | Trunk/extremities/chest wall | 0.37 (0.08–1.78) | 0.217 | 0.259 | 2.51 (0.46–13.74) | 0.287 | 0.268 |
| Institution (Stanford/BIDMC vs UCSF) | Stanford/BIDMC | 6.21 (2.12–18.18) | **<0.001** | **<0.001** | 1.95 (0.58–6.51) | 0.280 | 0.280 |
